# Pitfalls of using confocal-microscopy based automated quantification of synaptic complexes in honeybee mushroom bodies (*response to Peng and Yang 2016*)

**DOI:** 10.1038/s41598-017-09967-8

**Published:** 2017-08-29

**Authors:** Wolfgang Rössler, Johannes Spaethe, Claudia Groh

**Affiliations:** Behavioral Physiology & Sociobiology (Zoology II), Biozentrum, University of Würzburg, Am Hubland, 97074 Würzburg Germany

## Abstract

A recent study by Peng and Yang in *Scientific Reports* using confocal-microscopy based automated quantification of anti-synapsin labeled microglomeruli in the mushroom bodies of honeybee brains reports potentially incorrect numbers of microglomerular densities. Whereas several previous studies using visually supervised or automated counts from confocal images and analyses of serial 3D electron-microscopy data reported consistent numbers of synaptic complexes per volume, Peng and Yang revealed extremely low numbers differing by a factor of 18 or more from those obtained in visually supervised counts, and by a factor 22–180 from numbers in two other studies using automated counts. This extreme discrepancy is especially disturbing as close comparison of raw confocal images of anti-synapsin labeled whole-mount brain preparations are highly similar across these studies. We conclude that these discrepancies may reside in potential misapplication of confocal imaging followed by erroneous use of automated image analysis software. Consequently, the reported microglomerular densities during maturation and after manipulation by insecticides require validation by application of appropriate confocal imaging methods and analyses tools that rely on skilled observers. We suggest several improvements towards more reliable or standardized automated or semi-automated synapse counts in whole mount preparations of insect brains.

## Introduction

The mushroom bodies (MBs) of the insect brain perform high-level sensory integration and are involved in learning and memory formation^1–6^. Recent studies in the honeybee have shown that microglomerular synaptic complexes, termed microglomeruli (MG), in olfactory and visual input regions of the MB calyx express a high degree of structural synaptic plasticity during adult maturation^7–11^, in response to environmental factors during postembryonic development^7, 12^, after sensory exposure^13^, and following stable long-term memory formation^14, 15^. All of these studies used anti-synapsin labeling of relatively large presynaptic boutons of MG in the MB calyx, which, in some studies, was combined with f-actin phalloidin labeling of postsynaptic compartments or quantitative 3D serial electron microscopy analyses^10^. A recent review by Fahrbach and Van Nest^16^ provides a most comprehensive summary of these studies addressing possible links between brain plasticity and behavioral flexibility in social honeybees.

## Results and Discussion

The recent study by Peng and Yang^17^ used anti-synapsin immunolabeling and volume quantification of MB-calyx MG in whole-mount brains of adult honeybees to analyze synaptic maturation in the MBs under normal conditions and after larval treatment with sublethal dosages of imidacloprid, a neonicotinoid pesticide. Changes in the densities of synapsin-positive boutons from confocal image series were quantified to ask whether exposure of honeybee larvae to this insecticide alters densities and total numbers of MB synaptic boutons in adult brains which, as a consequence, might affect pollination behavior. Whereas the goal of this study addresses a very interesting and highly topical issue, the confocal-imaging based automated counting method for synapsin-positive boutons in MG of the MBs contains serious flaws. Although the authors claim they are aware of the difficulties of automated quantifications compared with visually guided counts by a human expert blind to the experimental treatment^18^, the discrepancies in an order of magnitude compared with previous analyses are not supported by any visual analyses to provide a confirmation of results from automated counts. In contrast, sample confocal images (Fig. 5 in ref. 17) indicate that MG numbers in olfactory and visual subregions of the MB calyx were significantly higher. This contrasts with the notion that “the results … were visually confirmed to ensure that all defined MGs in this diameter range were counted within the selected threshold range” (page 11 in ref. 17).

**Figure 1:**
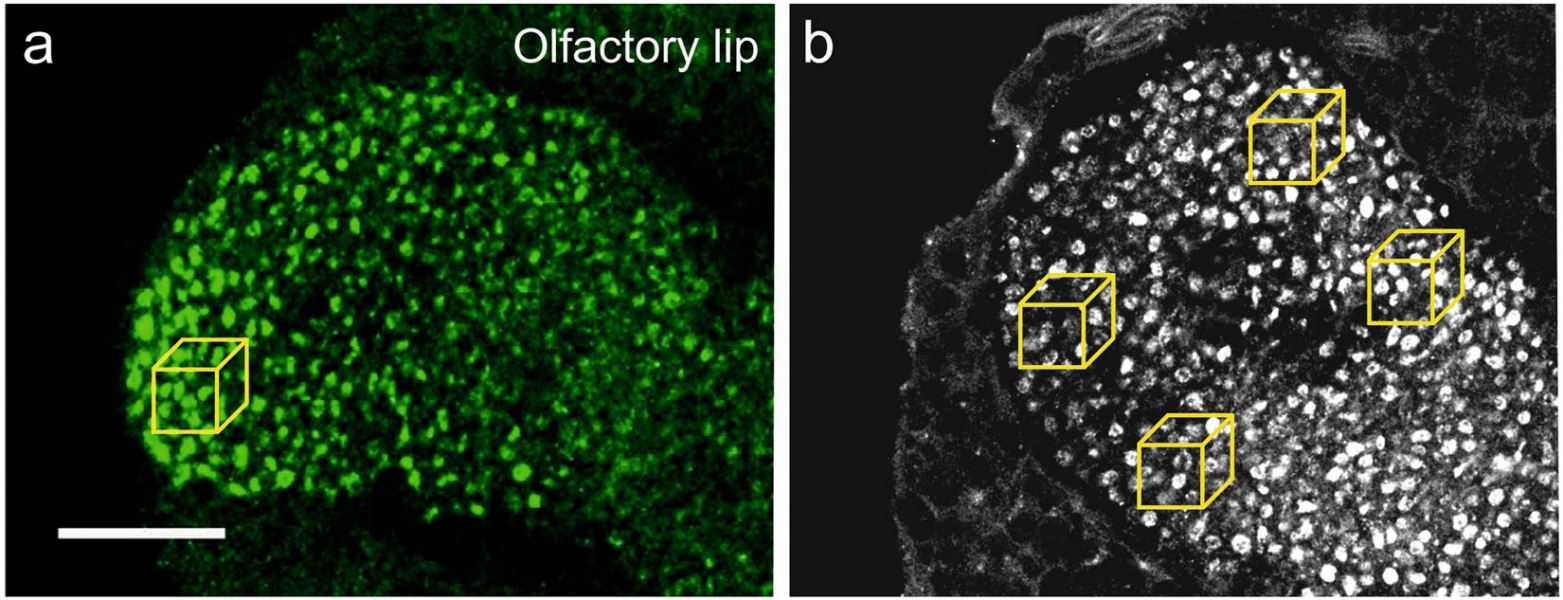
Comparison between confocal microscopy scans from two different studies. Synapsin immunolabeling of the brain of a 20-day old (**a**) and a 35-day old (**b**) honeybee worker, *Apis mellifera*. (**a**,**b**) Single frontal confocal section of the olfactory lip region showing the distribution of anti-synapsin labeled boutons. Each yellow box indicates a volume of 10 µm × 10 µm × 10 µm. (**a**) Detail view from Fig. 5D from Peng and Yang^17^ (the yellow box was added). (**b**) Detail view from Fig. 1b from Groh *et al*.^10^. In both cases the lip regions contain synapsin-positive boutons of similar size, distribution and density. However, automated counting in Peng and Yang^17^, on average, revealed 1.9 boutons per 1,000 µm^3^, whereas visually guided counts by Groh *et al*.^10^ quantified 32.9 MG per 1,000 µm^3^. Scale bar: A: 25 µm (also applies to B).

This is made most clear with Fig. 1 presenting a side-by-side comparison of images from the Peng and Yang study and from a rather exhaustive example of manual analysis by Groh *et al*.^10^, graphically demonstrating that despite the different counts, these tissues are largely similar and do closely resemble each other to a visual approximation, and therefore should have roughly comparable density numbers. The high density of synapsin-positive boutons visible in raw confocal images provided in the Peng and Yang paper (Fig. 5 in ref. 17) contrasts by an order of magnitude with the average numbers extracted from automated counts (Table 1 and 2 in ref. 17). This large discrepancy is further supported by comparison with data from previous studies using similar labeling techniques and visually guided semi-automated counts (Fig. 1; Table 1). Peng and Yang report extremely low MG densities that differ by a factor ~18 or more compared with the numbers extracted from visually guided counts obtained from multiple regions of interest throughout the MG calyx depth^10, 11, 15^. A most convincing proof of MG numbers was revealed by Groh *et al*.^10^ by showing that the density of synapsin-positive boutons in confocal image series was similar in 3D reconstructions of serial electron microscopy (EM) sections. The study by Groh *et al*.^10^, in general, greatly supports the reliability of visually guided MG counts obtained from fluorescently labeled synapsin-positive boutons in whole mount preparations. The obvious discrepancy of confocal imaging-based, automated volume counts by Peng and Yang is not supported by the confocal images they provided as the 1,000 µm^3^ tissue cube, even in a 2D view, clearly contains more than 0.8 to 2 synapsin-positive boutons (yellow boxes in Fig. 1, and Table 1; compare with Figure 5 and Table 1 and 2 in Peng and Yang^17^). For an average number of 1.46 MG per 1000 µm^3^ in the total MB calyx volume, the representative images presented in Fig. 5c–e (graphically suggesting MG densities of ~35 or more MG per 1000 µm^3^) could make up only a proportion of ~5% of the total MB calyx (as the average number is by a factor of approximately 20 lower than in the image shown). In consequence, 95% of the remaining MB calyx volume should be devoid of any MG if the average numbers reported by Peng and Yang were true. Earlier studies considered anatomical subcompartments in the MB calyx by sampling MG in selected volumes (regions of interest) and, for example, show that the MG distribution in the dense collar is very regular with densities around 65 MG/1000 µm^3^ and more heterogeneous with densities around 35 MG/1000 µm^3^ in the lip (Fig. 1b). The dense collar occupies up to 70% of the total collar, whereas the volume of the non-dense collar is substantially smaller^11^. Therefore, it appears reasonable and more informative to treat these anatomical subregions of the MB calyx separately for MG counts. In that line, from a neuroanatomical circuit perspective the differential effects after insecticide treatment even within subregions of the MB calyces reported by Peng and Yang are very difficult to interpret, and the results are not conclusive.

**Table 1:** Comparison of synapsin-positive bouton numbers between studies.

	Peng & Yang^17^	Krofczik *et al*.^8^	Wolschin *et al*.^9^	Groh *et al*.^10^		Muenz *et al*.^11^	Sommerlandt *et al*.^15^
forager age	unknown	37 days	5 days of foraging	35 days		32 days	unknown
quantification	automated counts			visually guided counts			
method	synapsin WM	synapsin thick section	synapsin WM	synapsin WM	serial EM	synapsin WM	synapsin WM
**Lip**							
total #/calyx	0.4 × 10^4^	NA	NA	15.8 × 10^4^	NA	19.1 × 10^4^	15.9 × 10^4^
#/1,000 µm^3^	1.9	358.7	~40.0*	32.9	24.5	35.6	36.3
volume (µm^3^)	2.2 × 10^6^	NA	NA	4.7 × 10^6^	NA	5.4 × 10^6^	4.6 × 10^6^
**Collar**							
total #/calyx	1.4 × 10^4^	NA	NA	30.5 × 10^4†^	NA	31.8 × 10^4†^	29.0 × 10^4†^
#/1,000 µm^3^	2.0	NA	~40.0*	65.9^†^	47.1^†^	63.0^†^	63.8^†^
volume (µm^3^)	7.2 × 10^6^	NA	NA	6.5 × 10^6^	NA	6.9 × 10^6^	4.8 × 10^6†^

Counts of synapsin-positive boutons in the mushroom body calyx lip and collar region of *Apis mellifera* foragers compiled from different studies. The density of boutons per 1,000 µm^3^ ranged in the (olfactory) lip region between 25 and 36 in visually guided counts from confocal image series or serial 3D electron microscopy analyses, and between 1.9 and 360 in studies using automated counts based on confocal image series. Similar differences between visually guided and automated counts are evident in the (visual) collar region. Whereas automated counts by Peng and Yang^17^ are at least ~18 times lower, the study by Krofczik *et al*.^8^ revealed up to ~10 times higher numbers compared to visually guided counts from confocal image series and serial electron microscopy. EM = electron micrograph, NA = not applicable, WM = whole mount. *lip and collar not differentiated, ^†^only dense collar region.

What may be the cause for the extremely low MG counts obtained by Peng and Yang? We think that the main source for this difference mainly resides in the misapplication of confocal microscopy followed by misevaluation of 3D confocal data stacks using automated image analysis. The methods section (page 10) lists a 40x and a 63x objective with working distances of only 0.1 mm - both objectives do not allow complete scans through the entire depth of the MB calyx (which is 550–600 µm in adult honeybees, cover slip and liquid not included; for comparison see Fig. 1D–E in ref. 11). Only the 20x objective listed in the methods section (working distance 0.59 mm) might allow a partial or nearly full scan of the MB calyx along the Z-axis, but due to optical physics constraints, a 20x/0.7 Ap DRY objective with its insufficient Z-axis scanning resolution and the application of 5 µm scanning intervals clearly does not provide the optical resolution required for reliable detection of individual synapsin-labeled synaptic boutons with diameters in the range of only 2 µm^10, 11^. It appears very likely that insufficient optical resolution combined with extremely weak fluorescence signals at tissue depths beyond 150 µm and the subsequent unsupervised application of image analysis tools are the cause of the reported extremely low MG counts. Unfortunately, the study by Peng and Yang lacks any data on checks comparing raw confocal data stacks with extracted automated counts routinely verifying what was resolved by confocal imaging at deeper layers and what was extracted by the automated image analysis or discarded.

Peng and Yang^17^ claim that their study is the “…first to observe a complete standard trend of the complete structure of the calyces of the adult bee”. This is not entirely true, as age-related changes of MG densities during normal adult maturation in the honeybee were recently reported in great detail by Muenz *et al*.^11^. Most importantly, however, the results on age-related changes in MG bouton densities reported by Peng and Yang^17^ largely contrast with several earlier studies including serial EM analyses^10, 11, 13^ showing that mature honeybee foragers have lower MB-calyx MG densities than younger bees (pruning effect). This discrepancy with earlier studies was also pointed out by Fahrbach and Van Nest^16^, but is largely ignored in the study by Peng and Yang^17^. In summary, we suggest that in the light of the serious methodological flaws outlined above the results by Peng and Yang on both maturation under normal conditions and after insecticide treatment should be viewed as preliminary and potentially flawed rather than conclusive.

Due to the striking discrepancies between the results from automated counts of synaptic complexes and those from visually guided counts, we compared the study by Peng and Yang^17^ with two other previous studies in the honeybee using automated MG counts based on confocal imaging^8, 9^ (Table 1). In contrast to Peng and Yang^17^, Krofczik *et al*.^8^ used thick agarose-embedded brain slices double labeled with anti-synapsin antibodies combined with f-actin phalloidin labeling, similar as introduced earlier by Groh *et al*.^7^. Normalizing the data by Krofczik *et al*.^8^ to 1,000 µm^3^ tissue cubes reveals extremely high numbers of ~360 and more MG per 1,000 µm^3^ in the olfactory subregion (lip) of the MB calyx (Table 1). This is in stark contrast with both the automated counts by Peng and Yang^17^ (~1.9 or less MG per 1,000 µm^3^) and the results from visually guided counts (~25–36) in all previous studies including 3D serial EM quantification for the MB lip region^10, 11, 15^ (Table 1). With ~40 synapsin-positive boutons per 1,000 µm^3^, automated counts by Wolschin *et al*.^9^ come closest to the results revealed by visually guided counts of other studies and greatly differ from the low numbers revealed Peng and Yang (Table 1) and the extremely high numbers from Krofczik *et al*.^8^. Interestingly, in contrast to the differences in synapsin-positive bouton counts, the overall volumes revealed for the MB calyx are largely similar across these studies (Table 1) indicating that potential differences in histochemical treatments did not affect the overall volumes.

Automated counts in the studies by Peng and Yang^17^, Krofczik *et al*.^8^ and Wolschin *et al*.^9^ were primarily based on thresholding and particle count algorithms. This strongly suggests that potential misapplication of confocal imaging and the subsequent misuse of automated image analysis algorithms caused the very low counts in Peng and Yang’s study. Our own attempts to use the thresholding and particle count tools from ImageJ (ImageJ 1.49 v; Wayne Rasband, NIH, Bethesda, MD), so far, have failed to produce reliable results compared with visually guided counts, even in image stacks at high optical resolution (for examples of image stacks see Supplementary data S1 and S2). We agree with Peng and Yang^17^ that using the human eye and expertise as a tool for synapse identification, as earlier suggested by Busse and Smith^18^, is difficult and time consuming, especially in analyzing large data sets. However, it is essential to perform checks in different MB calyx subregions and depths using the human eye and expertise to confirm data from automated image analysis by visual inspection of confocal data sets.

How can the comparability of MG counts in the MB calyx be improved in general? The MG in the MB calyces of the honeybee are delicate structures as the diameter of synapsin immunoreactivity is in the range of ~2 µm^10^, which comes close to the maximum resolution of classical confocal microscopy in the z-axis (see supplementary data S1 and S2). The optical resolution critically depends on the magnification and aperture of the objective and on confocal settings, in particular pinhole, gain, laser power, working distance, and intensity compensation in large Z-stacks. Groh *et al*.^10^ confirmed the size of projection neuron synaptic boutons in olfactory and visual subregions of the MB calyx of the honeybee using serial electron-microscopy based 3D reconstructions. However, fluorescent background staining and partly irregular shapes of olfactory and visual MG may further complicate quantitative confocal microscopy based measurements of synapsin-positive MG boutons^16, 19^. Furthermore, as the laser energy at high confocal magnification is focused on a small volume, this may easily result in substantial bleaching of fluorophores within a small tissue block, especially when obtaining large 3D image stacks. Altogether, this requires careful adjustments of the laser energy along the Z-axis and a fast scanning speed to compensate bleaching. Without these precautions, data may already be variable at the level of raw confocal images, especially in deeper tissue layers, which potentially represents the main reason for the weak fluorescence in Fig. 5b,f–h in Peng and Yang^17^. For potential improvement, the imaging depth can be increased and photo damage minimized by using two photon imaging.

Another approach for improvement is using double staining techniques to strengthen the selection criteria for individual MG in automated counts. Combining anti-synapsin immunostaining with f-actin phalloidin labeling has been introduced to better define MG borders as this combination of markers visualizes both pre- and postsynaptic compartments of individual MG^7, 20^. So far, however, this method worked only in thick agarose sections, and our own attempts to use it in whole mount preparations have failed because fluorescently labeled phalloidin gets washed out during the dehydration procedure by organic solvents. This asks for alternative tissue clearing agents and for a search for further molecular candidates and antibodies labeling distinct synaptic compartments. A dual criterion for the detection of synaptic units, however, should greatly enhance quantification by automated image analyses. Finally, image-processing tools are constantly improving, in particular the development of intelligent (or trainable) image analysis algorithms. This represents a great future potential for enhancement of automated image analyses in large data sets after various treatments like manipulations by environmental, hormonal or genetic factors including the appropriate controls. To promote the development of such tools across laboratories working on the honeybee, we provide two high-resolution confocal image stacks in a central layer of the honeybee MBs (lip and collar) as supplementary data stacks (supplementary data S1 and S2) together with the description of the image parameters and a detailed immunohistochemistry protocol for anti-synapsin labeling in whole mount brain preparations (supplementary methods S3). The 3D image stacks are freely downloadable for testing new image analyses algorithms.

In the end, however, the best automated counting tool will still require some degree of visual confirmation by a human expert’s eyes^18^. Therefore, until replicated and validated, the results of Peng and Yang should be viewed as preliminary and potentially flawed rather than conclusive. Due to their characteristic shape, large size, and high levels of structural plasticity, synaptic complexes (microglomeruli) in the MB calyces of the honeybee and also various other species of bees and ants will continue to be highly promising candidates for quantitative manipulative studies on the adaptive flexibility of neuronal microcircuits and their role in behavioral plasticity in a social context.

## Methods

We provide two examples of high-resolution confocal image stacks from a central layer within the honeybee MBs (lip and collar) as 3D supplementary data stacks (supplementary data S1 and S2). Honeybee workers (*Apis mellifera carnica*) were taken from the institutional apiary of Zoology II, University of Würzburg. All steps including materials for brain dissection, immunohistochemistry and anti-synapsin immunolabelling in whole mount brain preparations were similar to refs 10 and 11 and are provided as a detailed protocol under supplementary information S3. Confocal-microscopy equipment, imaging settings, and parameters for both confocal image stacks are also listed in detail under supplementary information S3.

## Electronic supplementary material

Supplementary dataset S1

Supplementary dataset S2

Supplementary Information S3
