# Supplementary figures and images for: Pitfalls of using confocal-microscopy based automated quantification of synaptic complexes in honeybee mushroom bodies (response to Peng and Yang 2016)
Source: Sci Rep. 2017 Aug 29;7:9786. doi: 10.1038/s41598-017-09967-8 (PMC5575136; doi:10.1038/s41598-017-09967-8)

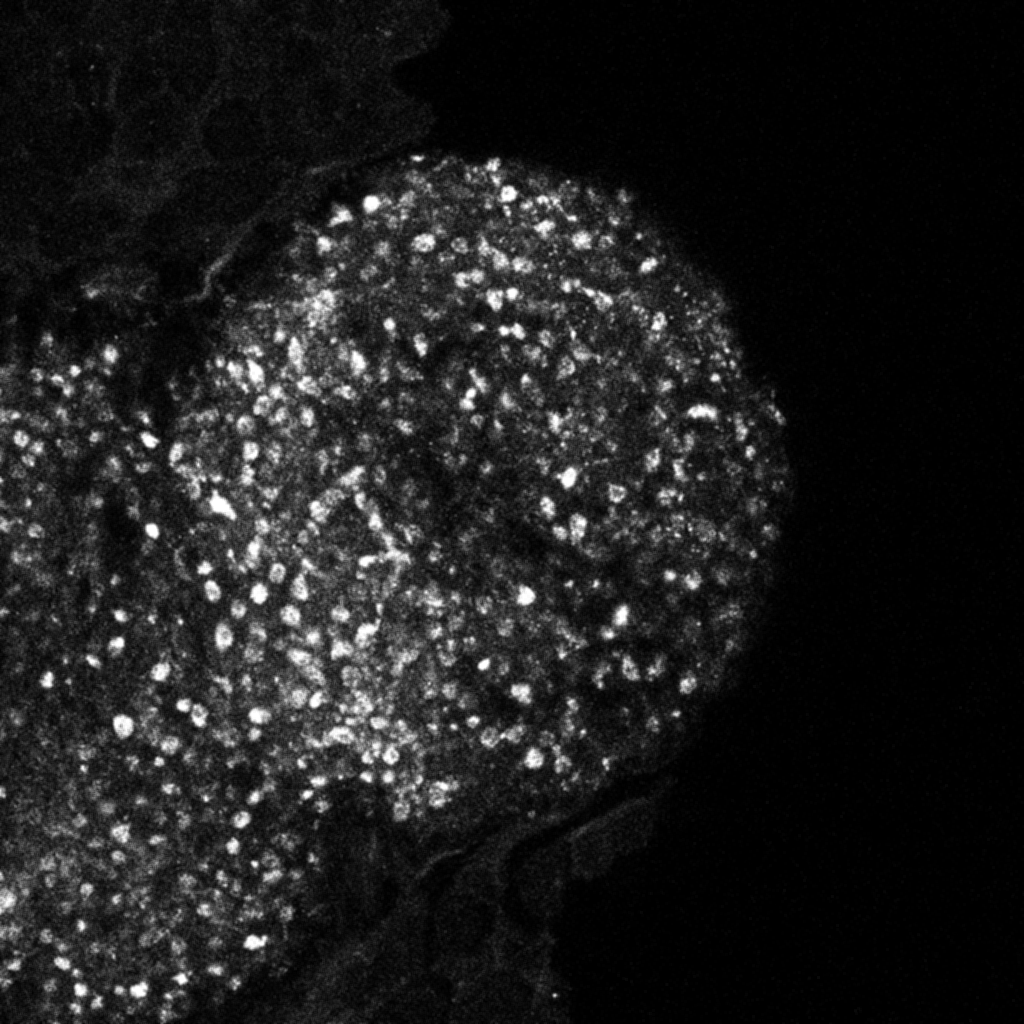

Supplement: Supplementary file 1 — Supplementary dataset S1 [file 41598_2017_9967_MOESM1_ESM.zip › Suplementary Data S1 Roessler_Spaethe_Groh.tif]

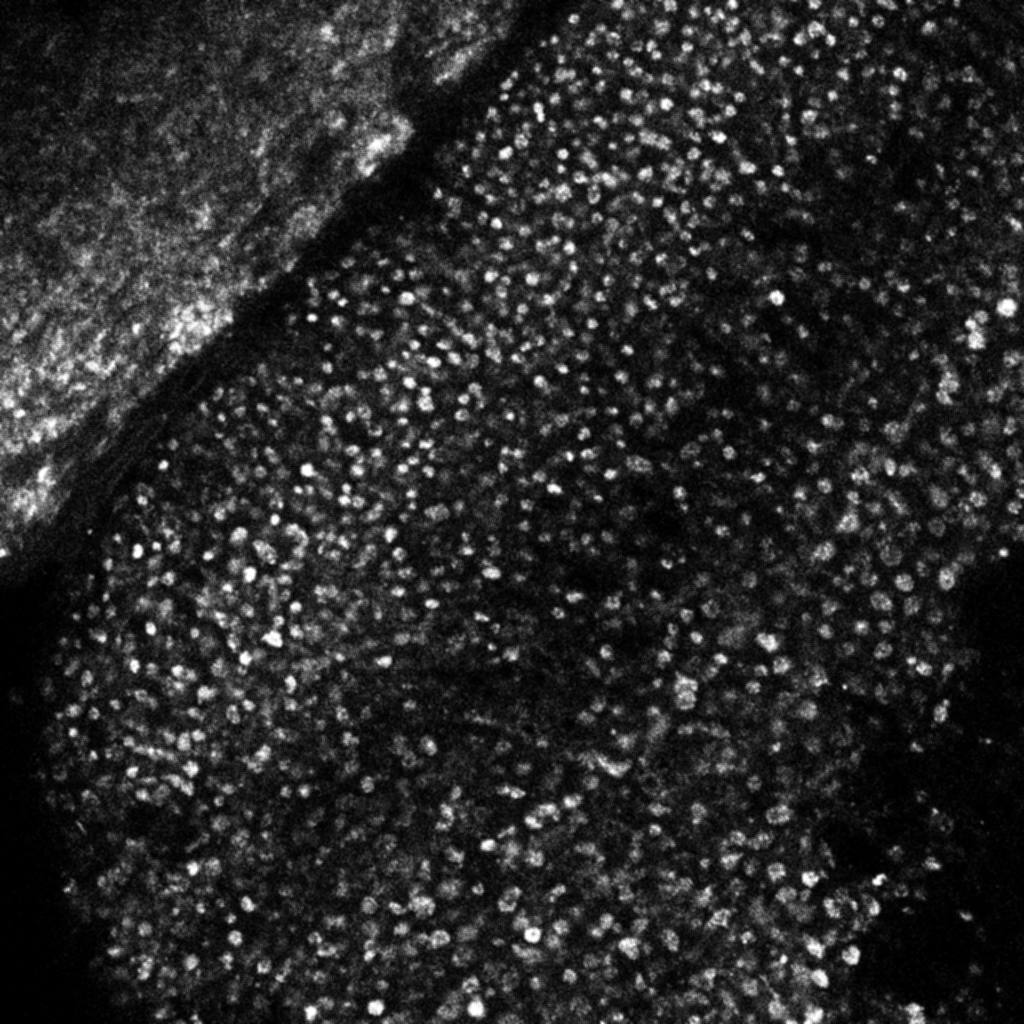

Supplement: Supplementary file 2 — Supplementary dataset S2 [file 41598_2017_9967_MOESM2_ESM.zip › Supplementary Data S2 Roessler_Spaethe_Groh.tif]
