# Supplementary material for: Pitfalls of using confocal-microscopy based automated quantification of synaptic complexes in honeybee mushroom bodies (response to Peng and Yang 2016)
Source: Sci Rep. 2017 Aug 29;7:9786. doi: 10.1038/s41598-017-09967-8 (PMC5575136; doi:10.1038/s41598-017-09967-8)
Supplement: Supplementary file 3 — Supplementary Information S3 [file 41598_2017_9967_MOESM3_ESM.pdf]

Pitfalls of using confocal-microscopy based automated quantification of synaptic complexes in honeybee mushroom bodies (*response to Peng and Yang 2016*)

Wolfgang Rössler*, Johannes Spaethe, Claudia Groh

Supplementary Information

Supplementary information: parameters of 3D image stacks (suppl. data S1 and S2)

Supplementary data S1: Confocal image stack *Apis mellifera* MB lip forager (32 days)
Supplementary data S2: Confocal image stack_*Apis mellifera*_MB collar forager (32 days)

Laser scanning confocal microscopy:

Leica TCS SP2 AOBS (Leica Microsystems AG, Wetzlar, Germany)
Objective: HC PL APO 63X/1.20 NA imm

Imaging Parameters:

Image sizes XY: 119.047619 µm X 119.047619 µm
Image format XY: TIFF 1024 x 1024 pixel
Pixel size XY: 0.116257 µm X 0.116257 µm
Z-axis scanning step size: 0.5 µm

Supplementary Methods S3

Protocol for anti Synapsin Immunolabeling of *Apis mellifera* Whole-Mount Brains
(modified from Groh et al. 2012; Muenz et al. 2015)

- Immobilize bee on ice or with CO_2
- Fix head with dental wax (Surgident, Sigma Dental Systems, Handewitt, Germany) in customized acrylic holders
- Cover the head entirely with ice cold physiological saline (130 mM NaCl, 5 mM KCl, 4 mM $MgCl_2$, 5 mM $CaCl_2$, 15 mM Hepes, 25 mM glucose, 160 mM sucrose; pH 7.2)
- Cut a rectangular window frontally into the head capsule
- Remove glands, tracheae and proboscis
- Decapitate the bee and immerse the head immediately overnight in ice cold 4% formaldehyde (FA, methanol free, 28908, Fischer Scientific, Schwerte, Germany) in 0.01 M phosphate-buffered saline (PBS; pH 7.2) at 4°C
- Dissect the brain from the head capsule under PBS, and carefully remove the retinae and ocelli
- Wash in PBS (5 x 10 min)
- Permeabilize the brain in 0.2%Triton X-100 (Tx) in PBS (2 x 10 min)
- Block the brain tissue in 2% normal goat serum (NGS; 005-000-121, Jackson ImmunoResearch Laboratories, West Grove, PA, USA) in 0.2% PBS-Tx for 1 h at room temperature
- Incubate the brain in the primary antibody SYNORF1 (1:50; available via Developmental Studies Hybridoma Bank, University of Iowa, USA) in 0.2% PBS-Tx with 2% NGS for at least four nights at 4°C
- Wash in PBS (5 x 10 min)
- Incubate the brain in CF488A goat anti-mouse secondary antibody (1:250; Biotium, CA, USA) in 1% NGS-PBS for four nights at 4°C
- Wash in PBS (5 x 10 min)
- Dehydrate the brain in an ascending ethanol series: 30%, 50%, 70%, 90%, 95%, 3 x 100%, 10 min for each step)
- Clear the brain in methyl salicylate (M-2047, Sigma Aldrich, Steinheim, Germany)
- Mount the brain in methyl salicylate using custom aluminum slides with a central hole covered from both sides by #00 cover slips (55-80 µm thick, 5812914, Fisher Scientific, Schwerte, Germany).
